# Clinical effect of intramuscular calcitonin compared with oral celecoxib in the treatment of knee bone marrow lesions: a retrospective study

**DOI:** 10.1186/s13018-020-01746-y

**Published:** 2020-06-23

**Authors:** Jiaming Zhou, Wuyi Xiong, Pengguo Gou, Zhao Chen, Xing Guo, Xiaoyang Huo, Yuan Xue

**Affiliations:** 1 Department of Orthopaedic Surgery, Tianjin Medical University General Hospital, Tianjin, 300052 China; 2 Tianjin Key Laboratory of Spine and Spinal Cord, Tianjin Medical University General Hospital, Tianjin, 300052 China; 3 Department of Orthopaedic Surgery, The Fifth People’s Hospital of Datong, Datong, 037006 China

**Keywords:** Calcitonin, Celecoxib, Bone marrow lesions, Osteoarthritis

## Abstract

**Background:**

Bone marrow lesions (BMLs) are a common finding in patients with osteoarthritis (OA), which are predictors of progression and pain related to cartilage damage in OA. The objective of the present research was to compare the short-term clinical effect of intramuscular calcitonin and oral celecoxib in treating knee BMLs.

**Patients and methods:**

Between January 2016 and December 2018, the medical records of patients with knee BMLs treated by intramuscular calcitonin or oral celecoxib were reviewed. Visual analog scale (VAS) and the Western Ontario and McMaster University Osteoarthritis Index (WOMAC) were used to assess knee pain and function, respectively. BMLs were assessed by MRI scans and were scored by the modified Whole-Organ MRI Score (WORMS). The safety of these two medications was also evaluated.

**Results:**

A total of 123 eligible patients who received calcitonin treatment (*n* = 66) or celecoxib treatment (*n* = 57) were included. All patients were followed up clinically and radiographically for 3 months. The VAS and WOMAC scores were lower statistically in calcitonin group than celecoxib group at 4-week and 3-month follow-up. For BMLs, the WORMS scores in the calcitonin group were significantly lower than the celecoxib group. Besides, statistically higher MRI improvement rates were found in the calcitonin group compared with the celecoxib group at 4-week follow-up (21.21% vs. 7.01%; *P* = 0.039) and 3-month follow-up (37.88% vs. 15.79%; *P* = 0.006).

**Conclusion:**

Intramuscular calcitonin 50 IU once daily demonstrated a better short-term effect for knee BML patients compared with oral celecoxib 200 mg twice per day.

## Background

Knee osteoarthritis (OA), the most common joint disease around the world, often leads to pain, stiffness, or even loss of function [1–5]. When patients suffer from knee pain, bone marrow lesions (BMLs) are a common finding in magnetic resonance imaging (MRI) and are characterized by a hyperintensity in the marrow in fat-suppressed T2-weighted images, which are a reversible but highly painful manifestation in patients with knee pain [6]. Felson et al. reported that patients with knee OA concomitant BMLs had a higher incidence of knee pain than those with a similar degree of radiographic knee OA but without BMLs [7]. It is well known that various diagnoses, especially degenerative arthritis, could contribute to BMLs [6, 8]. The exact pathogenetic processes and role of painful BMLs in OA knees are not currently known. For patients with this issue, the best treatment is still unclear.

Patients with knee OA often suffer joint pain and are usually treated using nonsteroidal anti-inflammatory drugs (NSAIDs), opioid analgesics, and intra-articular injection such as hyaluronic acid, corticosteroids, and platelet-rich plasma [9, 10]. Oral NSAIDs are the most frequently used pharmaceuticals for pain control and are usually recommended in the OA Clinical Practice Guidelines for the treatment of widespread OA to relieve pain [11–13]. However, because these drugs are related to gastrointestinal and cardiorenal toxicity and are ineffective in some patients, it is necessary to develop specific and efficacious drugs for patients with knee OA suffering from pain.

Calcitonin is an anti-osteoclastic drug and can effectively inhibit the bone resorption of osteoclasts and has been proved to be effective in osteoporosis and other diseases which involved accelerated bone turnover [14, 15]. It could significantly antagonize subcartilage bone changes, alleviate cartilage degradation, and prevent the net loss of collagen, hyaluronic acid, and proteoglycan in OA animal models [16–18]. In patients with OA, calcitonin was found to improve dysfunction, reduce cartilage degeneration, and reduce the level of biomarkers for bone resorption and cartilage degeneration [19–21]. Meanwhile, in a microarray analysis, increased bone turnover was found in BMLs [22]. Hence, calcitonin may have potential as a method for treating knee BMLs. To our knowledge, the effect of calcitonin in knee BMLs has not been investigated in previous study. Celecoxib was chosen as the comparator because celecoxib is superior to other NSAIDs owing to a lower incidence of gastrointestinal side effects [23]. In addition, celecoxib is effective in the treatment and wide application of knee OA which can improve pain, movement, and quality of life [24]. OA is a main cause of disability and impairs quality of life in older adults, particularly when the knee is affected [25]. Many patients imminently hope to relieve symptoms as soon as possible in order to reintegrate into society. We hypothesized that intramuscular calcitonin had better short-term clinical effect compared with oral celecoxib for knee BMLs. Hence, the current research aimed to explore the clinical effect of intramuscular calcitonin compared with oral celecoxib in treating knee BML patients.

## Patients and methods

### Patients

**Fig. 1:**
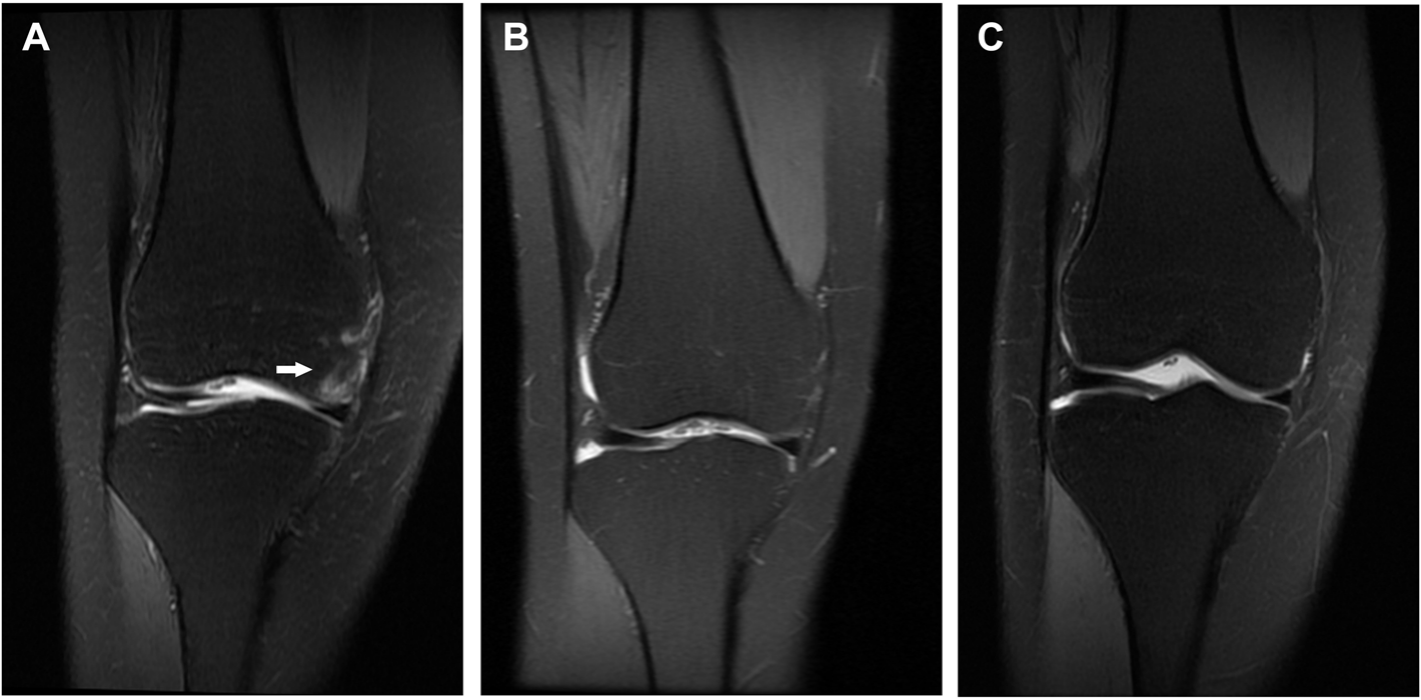
A patient with BMLs (arrow) on MRI: hyperintensity on fat-suppressed T2-weighted image (**a**). After calcitonin treatment, no abnormal signal intensity was found at 4-week follow-up (**b**) and 3-month follow-up (**c**). *BMLs* bone marrow lesions, *MRI* magnetic resonance imaging

We reviewed 152 consecutive patients in our hospital who were diagnosed with early- to mid-stage knee OA and BMLs (Fig. 1a) between January 2016 and December 2018 according to the diagnostic criteria of the American College of Rheumatology [26, 27].

The severity of OA was evaluated by Kellgren-Lawrence (K-L) grading score through X-ray. The BMLs were defined as an alteration in bone marrow signal intensity, with ill-defined hyperintensity in fat-suppressed T2-weighted MRI images [28–30]. Using the modified Whole-Organ MRI Score (WORMS), BMLs were classified as 0–3 using the largest percentage of bone area in the entire subregion (three lesion subregions: anterior, central, and posterior, and there was an additional subregion of the tibia representing the subspine of the tibia) as follows: 0, none; 1, 1–25% of the subregion; 2, 26–50% of the subregion; 3, 51–100% of the subregion [31]. The total scores of the knee BMLs were obtained by adding the BML scores of all sites, and the total score range of the knee BMLs was 0–45. BML scores ≥ 1 in any subregion was defined as the presence of BMLs in the entire knee.

Patients who had the following characteristics were excluded: (1) bilateral knee OA (*n* = 11) and (2) lack of adequate follow-up data (*n* = 18). Finally, 123 patients were included in this study. All the patients had early- to mid-stage knee OA and BMLs, with severity grade 1–3 according to Kellgren-Lawrence (K-L) grading score.

### Treatment

One hundred and twenty-three patients were retrospectively studied. Both medications were prescribed by two senior physicians (YX and PG). YX prescribed intramuscular calcitonin for patients if they meet the indications as follows: knee OA with severity grade 1–3 according to K-L grading score on X-ray and BMLs on MRI. PG routinely prescribed celecoxib for patients with grade 1–3 knee OA, no matter whether BMLs existed on MRI. A total of 66 patients received an intramuscular injection of 50 IU of calcitonin (Miacalcic®, Novartis Pharma Schweiz AG, Rotkreuz, Switzerland) once daily, and 57 patients received 200 mg of celecoxib (Celebrex®, Pfizer Inc., New York, USA) orally twice daily for 4 weeks. In addition, all patients received the same basic treatment including health education, physical therapy, and weight-bearing restrictions. Unloader brace, calcium, or vitamin D supplements were not given to any patients. Baseline data for all patients, including age, gender, smoking, body mass index (BMI), and knee OA image data, were collected from clinical records. At each follow-up, patients’ data were collected.

### Post-intervention assessment

The post-intervention assessment included knee pain and function using the VAS and the Western Ontario and McMaster University Osteoarthritis Index (WOMAC) scores and adverse events, which were recorded at baseline and at 1, 2, and 4 weeks and 3 months after treatment. VAS scores ranged from 0 to 10 for the assessment of knee pain perception [32]. WOMAC is a validated survey including three domains, namely joint pain (0–20), stiffness (0–8), and the limitation of physical function (0–68) [33].

### Evaluation of imaging data

Four weeks and three months after treatment, patients were asked to undergo a knee MRI. All studies were performed in one center on the same equipment (Discovery MR750, General Electric, Milwaukee, Wisconsin, USA). Using WORMS, the BMLs were scored. The changes in the BML scores were calculated by subtracting the follow-up total knee BML scores from the total knee BML scores at baseline. A decrease in BML scores of more than 1 was considered to be an effective improvement [34].

Two researchers (JZ and WX) collected and reviewed all of the imaging data. To guarantee data reliability, they held regular meetings and discussions. The intraclass correlation coefficients of intraobserver and interobserver reproducibility for WORMS were 0.94 and 0.90, respectively. MRI was performed at baseline and 4-week and 3-month follow-up. In addition, changes and scores for the BMLs were assessed. One patient with BMLs treated using calcitonin is illustrated in Fig. 1. The BML improvement rate was also calculated.

### Power analysis

Based on previous studies and our pilot experiment, we assumed normal distribution and a VAS standard deviation (SD) of 0.8. With a two-sided *α* = 0.05, a sample size of 42 patients in each group gave a power of 0.8 to detect a mean difference of 0.5 in VAS.

### Statistical analysis

All results are expressed as mean values ± standard deviation or frequencies (with proportions). Differences between groups of continuous variables and categorical variables were analyzed by independent sample *t* test and chi-square test, respectively. Intragroup differences were analyzed using a paired *t* test. SPSS 21.0 (IBM Corp, Armonk, NY, USA) was performed to analyze all of these data, and a *P* value less than 0.05 was accepted to be statistically significant.

## Results

### Baseline characteristics

A total of 123 patients (69 men and 54 women) were enrolled with a mean ± SD age of 60.47 ± 9.54 years, and a mean BMI of 25.84 ± 4.83 kg/m^2^. According to the K-L criteria, 21 patients were grade 1, 44 patients were grade 2, and 58 patients were grade 3. No statistical differences were found between the two groups in age, gender, smoking, BMI, VAS, WOMAC, and BML scores (*P* > 0.05) (Table 1).

**Table 1:** Baseline characteristics of patients

Characteristics	Calcitonin (*n* = 66)	Celecoxib (*n* = 57)	*P* value
Age, mean (years) ± SD	59.49 ± 9.32	61.54 ± 8.48	0.620
Sex, *n* (male) (%)	38 (57.58)	31 (54.39)	0.722
Smokers, *n* (%)	24 (36.36)	21 (36.84)	0.956
BMI, mean (kg/m^2^) ± SD	26.38 ± 4.12	25.31 ± 5.50	0.481
Pain VAS scores (0–10)	6.48 ± 1.08	6.52 ± 0.87	0.876
WOMAC scores			
Total scores (0–96)	54.09 ± 10.24	54.60 ± 10.88	0.878
Pain scores (0–20)	12.08 ± 4.09	12.15 ± 2.62	0.943
Stiffness scores (0–8)	4.95 ± 0.97	4.90 ± 0.70	0.857
Physical function scores (0–68)	37.07 ± 9.54	37.54 ± 11.31	0.883
Kellgren-Lawrence criteria, *n* (%)			
Grade 1	11 (16.67%)	10 (17.54%)	0.986
Grade 2	24 (36.37%)	20 (35.09%)	
Grade 3	31 (49.96%)	27 (47.37%)	
BMLs scores	4.90 ± 1.35	4.95 ± 1.08	0.856

*BMI* body mass index, *VAS* visual analog scale, *WOMAC* Western Ontario and McMaster Universities Osteoarthritis Index, *BMLs* bone marrow lesions

### Clinical outcome

Symptoms of the disease decreased over time in both therapeutic groups, and differences between the VAS scores for pain and the WOMAC scores in both groups are shown in Table 2. Both calcitonin and celecoxib had an onset of response and provide pain relief and improvement of stiffness and physical function. The VAS and WOMAC scores were significantly improved compared with the baseline data in the two groups. Moreover, the VAS and WOMAC scores in the calcitonin group decreased more significantly compared with the celecoxib group.

**Table 2:** Comparison of clinical outcomes between two groups

Characteristics	Calcitonin (*n* = 66)	Celecoxib (*n* = 57)	*P* value
Pain VAS scores (0–10)			
Baseline	6.48 ± 1.08	6.52 ± 0.87	0.876
1 week	4.95 ± 1.28*	5.76 ± 0.89*	0.022
2 weeks	3.71 ± 0.96*	4.62 ± 0.92*	0.003
4 weeks	2.95 ± 0.80*	3.57 ± 0.87*	0.021
3 months	1.86 ± 0.65*	2.57 ± 0.81*	0.008
WOMAC scores			
Total scores (0–96)			
Baseline	54.09 ± 10.24	54.60 ± 10.88	0.878
1 week	41.00 ± 8.84*	49.89 ± 10.45	0.005
2 weeks	35.07 ± 7.43*	44.24 ± 10.10*	0.002
4 weeks	31.45 ± 6.67*	39.80 ± 8.63*	0.001
3 months	26.92 ± 6.81*	36.01 ± 7.93*	< 0.001
Pain scores (0–20)			
Baseline	12.08 ± 4.09	12.15 ± 2.62	0.943
1 week	8.89 ± 3.25*	10.87 ± 2.41	0.031
2 weeks	7.57 ± 2.53*	9.82 ± 2.06*	0.003
4 weeks	6.82 ± 2.19*	8.48 ± 1.80*	0.011
3 months	5.78 ± 1.55*	7.70 ± 1.63*	< 0.001
Stiffness scores (0–8)			
Baseline	4.95 ± 0.97	4.90 ± 0.70	0.857
1 week	4.10 ± 0.89*	4.62 ± 0.67	0.037
2 weeks	3.29 ± 0.90*	3.95 ± 0.86*	0.019
4 weeks	2.67 ± 0.86*	3.62 ± 0.67*	0.001
3 months	2.00 ± 0.84*	2.95 ± 0.92*	< 0.001
Physical function scores (0–68)			
Baseline	37.07 ± 9.54	37.54 ± 11.31	0.883
1 week	28.02 ± 8.11*	34.40 ± 11.00	0.039
2 weeks	24.21 ± 7.15*	30.48 ± 9.47*	0.020
4 weeks	21.96 ± 6.44*	27.70 ± 9.04*	0.023
3 months	19.14 ± 6.84*	25.35 ± 8.39*	0.012

*VAS* visual analog scale, *WOMAC* Western Ontario and McMaster Universities Osteoarthritis Index

*P < 0.05 vs baseline

### BML imaging data

The patients between the two groups showed a similar BML scores at baseline (*P* > 0.05) (Table 1). Based on MRI results, significantly lower BML scores were found in the calcitonin group compared with the celecoxib group at 4-week follow-up (4.28 ± 1.41 vs. 4.83 ± 1.01) and 3-month follow-up (3.63 ± 1.55 vs. 4.35 ± 1.22) (Table 3). In addition, BML changes scores in the calcitonin group showed statistical improvements compared with the celecoxib group. MRI examination results of the two groups also showed the patients in the calcitonin group had higher MRI improvement rate at 4-week follow-up (21.21% vs. 7.01%; *P* = 0.039) and 3-month follow-up (37.88% vs. 15.79%; *P* = 0.006) (Table 4).

**Table 3:** BMLs scores changed between two groups

	Original values			Changes		
	Calcitonin (*n* = 66)	Celecoxib (*n* = 57)	*P* value	Calcitonin (*n* = 66)	Celecoxib (*n* = 57)	*P* value
Baseline	4.90 ± 1.35	4.95 ± 1.08	0.856			
4 weeks	4.28 ± 1.41*	4.83 ± 1.01	0.049	0.62 ± 1.15	0.12 ± 0.46	0.014
3 months	3.63 ± 1.55*	4.35 ± 1.22*	0.005	1.27 ± 1.50	0.60 ± 1.17	0.028

*BMLs* bone marrow lesions

*P < 0.05 vs baseline

**Table 4:** Percentage of patients with improved BMLs

	Calcitonin (*n* = 66)	Celecoxib (*n* = 57)	*P* value
4 weeks	14 (21.21%)	4 (7.01%)	0.039
3 months	25 (37.88%)	9 (15.79%)	0.006

*BMLs* bone marrow lesions

### Adverse events

Seven patients (10.61%) in the calcitonin group and six patients (10.53%) in the celecoxib group reported at least one adverse event, with no significant differences (*P* = 0.966). Diarrhea (1; 1.52%), nausea (1; 1.52%), headache (2; 3.03%), hot flushes (3; 4.55%), and hypocalcemia (2; 3.03%) were reported in the calcitonin group. Gastroesophageal reflux disease (2; 3.51%), hypodynamia (3; 5.26%), headache (2; 3.51%), and dizziness (1; 1.75%) occurred in the celecoxib group.

## Discussion

OA is the most common progressive joint disease and responsible for pain, disability, debilitation, and socioeconomic cost worldwide [1–5]. We observed that for knee BMLs, 50 IU of calcitonin intramuscularly per day had a more beneficial effect than oral administration of 200 mg twice per day of celecoxib. The lower VAS and WOMAC scores were found in the calcitonin group which had lower BML scores and higher MRI improvement rates based on MRI examination results at 4-week and 3-month follow-up compared with the celecoxib group. The current study showed the same result that a decrease in BMLs reduced the pain which was in line with several studies [7, 8, 30, 31], and treatment of osteoarthritis might benefit from a lesion-specific therapeutic approach by reducing the area of BMLs [35].

BMLs, representing focal bone remodeling because of excessive loading, are predictors of progression and pain related to cartilage damage in OA [34, 36]. A decreased risk of cartilage loss was associated with the absence of BMLs, and progressive and new BMLs presented a higher risk of cartilage loss in the same subregion [37]. BMLs are also closely related to dysplasia of the affected side which increases the risk of structural progression in knee OA [8, 28]. In addition, Felson and coworkers founded that BMLs markedly increase the risk for structural deterioration and an increase in BMLs was related to the development of knee pain [28, 34]. Celecoxib, one of the NSAIDs which selectively inhibit COX-2, has been extensively used in the treatment of chronic pain such as osteoarthritis and rheumatoid arthritis [38]. However, celecoxib was found to have a neutral effect similar to placebo on cartilage volume loss in knee OA patients [39], and for patients with knee OA, a randomized controlled trial using MRI shown no difference between celecoxib and placebo in the progression of cartilage volume loss [40].

Calcitonin is a natural polypeptide that acts on specific receptors to effectively inhibit osteoclast function [41]. The significant protective effect of calcitonin in OA cartilage has been shown by previous studies [17, 18, 42, 43]. Furthermore, salmon calcitonin has been shown to resist cartilage degradation and resist bone resorption in the OA knee [7, 44]. Therefore, calcitonin has a dual protective effect in cartilage and subchondral bone and could be used in knee BMLs. In this research, all the enrolled knee OA patients demonstrated BMLs and significant improvement rates of BMLs were found in the calcitonin group compared with the celecoxib group. Besides, calcitonin has already been shown to have a beneficial effect on BMLs such as calcitonin has been used by a few researchers to treat hip BMLs [45, 46] and has been confirmed to provide a beneficial effect in lumbar BMLs, also known as type I Modic changes [47]. In addition, cartilage lesions are a common type of joint pathologic change in OA. The characteristics of chondroextracellular matrix imbalance include loss of matrix components and an increase in matrix degradation protease levels, which plays a vital part in the evolution of OA [48, 49]. However, it should be noted that in both groups, the pain and function improvements were gradually relieved. This phenomenon in the celecoxib group was unusual because celecoxib is a systemic rapid onset drug for OA, with the maximum pain-relieving effect at 2–4 weeks (mean 2.3 weeks) [50, 51]. One possible reason is that the pain of OA is complicated and could originate from BMLs, synovitis, effusions, periarticular lesions, and bursitis [52]. In this study, all patients demonstrated BMLs on MRI, and hence, the major cause of pain may originate from BMLs for these patients. The local high turnover of BMLs has been confirmed [53, 54], which might explain why anti-absorbents such as calcitonin could play an effective role on the extensive symptoms related with the lesions [55]. However, few literatures reported the efficacy of celecoxib in BMLs. We suppose that the effect of celecoxib in BMLs might be relatively insensitive and the time of maximal effect was delayed. Nevertheless, the mechanisms of pain in BMLs and the effect of calcitonin and celecoxib in this condition still need to be studied.

The current study had some limitations. This study is limited by the lack of randomization, which might lead to potential bias. Besides, a placebo group was not included in this research because all eligible patients were diagnosed by two physicians, one of whom treated patients with calcitonin and the other with celecoxib. In addition, although the MRI was performed on the same equipment and high intra- and inter-observer reliability of WORMS were found, the MRI protocol was not standardized. Moreover, the sample size in this study was not large enough. Further randomized placebo-controlled studies are needed to evaluate the clinical effect of calcitonin and celecoxib in BMLs.

## Conclusion

In summary, the results of the present study indicated that compared with celecoxib, intramuscular calcitonin provided a greater short-term clinical efficacy in the treatment of knee BMLs patients.

## Data Availability

Please contact the author for data requests.

## Abbreviations

BMI: Body mass index
BMLs: Bone marrow lesions
MRI: Magnetic resonance imaging
NSAIDs: Nonsteroidal anti-inflammatory drugs
OA: Osteoarthritis
VAS: Visual analog scale
WOMAC: Western Ontario and McMaster Universities Osteoarthritis Index
WORMS: Whole-Organ MRI Score
